# Case Report: Acquired Generalized Anhidrosis Caused by Brain Tumor: Review of the Literature

**DOI:** 10.3389/fendo.2022.877715

**Published:** 2022-05-26

**Authors:** Kohei Kawahara, Yasuto Shimomura, Yuichiro Oshima, Tadashi Watanabe, Toshinori Hori, Akihisa Okumura, Hideyuki Iwayama

**Affiliations:** ^1^Department of Pediatrics, Aichi Medical University School of Medicine, Nagakute, Japan; ^2^Department of Dermatology, Aichi Medical University School of Medicine, Nagakute, Japan; ^3^Department of Neurosurgery, Aichi Medical University School of Medicine, Nagakute, Japan

**Keywords:** anhidrosis, sweating, hypothalamus, brain tumor, germinoma, panhypopituitarism

## Abstract

**Purpose:**

There has been limited focus on sweating failure in patients with brain tumor. We report two patients with generalized anhidrosis caused by germinoma. We also review previous reports of generalized anhidrosis due to brain tumor.

**Case Reports:**

Patient 1 was a 12-year-old boy with repetitive heat shock-like episodes even in winter. Based on Minor’s test, he was diagnosed with generalized anhidrosis. Magnetic resonance imaging (MRI) revealed the absence of high signal intensity of the posterior pituitary. He was initially diagnosed with central diabetes insipidus. However, an MRI scan performed after 3 months revealed an enlarged pituitary stalk. He was finally diagnosed with germinoma by pituitary biopsy. After chemotherapy and radiation, sweating was partially resolved. Patient 2 was a 12-year-old girl with growth hormone deficiency and generalized anhidrosis. She was diagnosed with germinoma based on MRI and pituitary biopsy findings. After chemotherapy and radiation, the sweating resolved completely.

**Discussion:**

In our literature search, we identified four patients with anhidrosis due to brain tumor, including our cases. All patients had germinoma and continued to require hormone replacement therapy after treatment of germinoma. Two patients with incomplete recovery of sweating had the involvement in the hypothalamus, whereas one patient with complete recovery showed a lack of evident hypothalamic involvement. Improvement in sweating in one patient was not described.

**Conclusion:**

Germinoma can cause anhidrosis, and involvement in the hypothalamus may be relevant to incomplete recovery of sweating.

## 1 Introduction

The center of thermoregulatory sweating is located in the medial preoptic areas of the hypothalamus (1). Damage to the sweating center results in generalized anhidrosis. Generalized anhidrosis is determined using a thermoregulatory sweat test such as Minor’s starch iodine test (Minor’s test). Acquired generalized anhidrosis can arise from systemic autoimmune diseases and central nervous system disorders such as brain tumor. There has been limited research focus on sweating failure as the initial presentation in patients with brain tumor. Evaluation based on sympathetic nerve activity showed postoperative impairment of sweating function in patients with suprasellar tumors such as craniopharyngioma (2). When the cause of acquired generalized anhidrosis is unknown, it is classified as acquired idiopathic generalized anhidrosis (AIGA) (3).

Herein, we report two patients with generalized anhidrosis caused by germinoma. We also performed a literature review of generalized anhidrosis caused by brain tumor.

## 2 Subjects and Methods

### 2.1 Cases Presentation

#### 2.1.1 Patient 1

**Figure 1:**
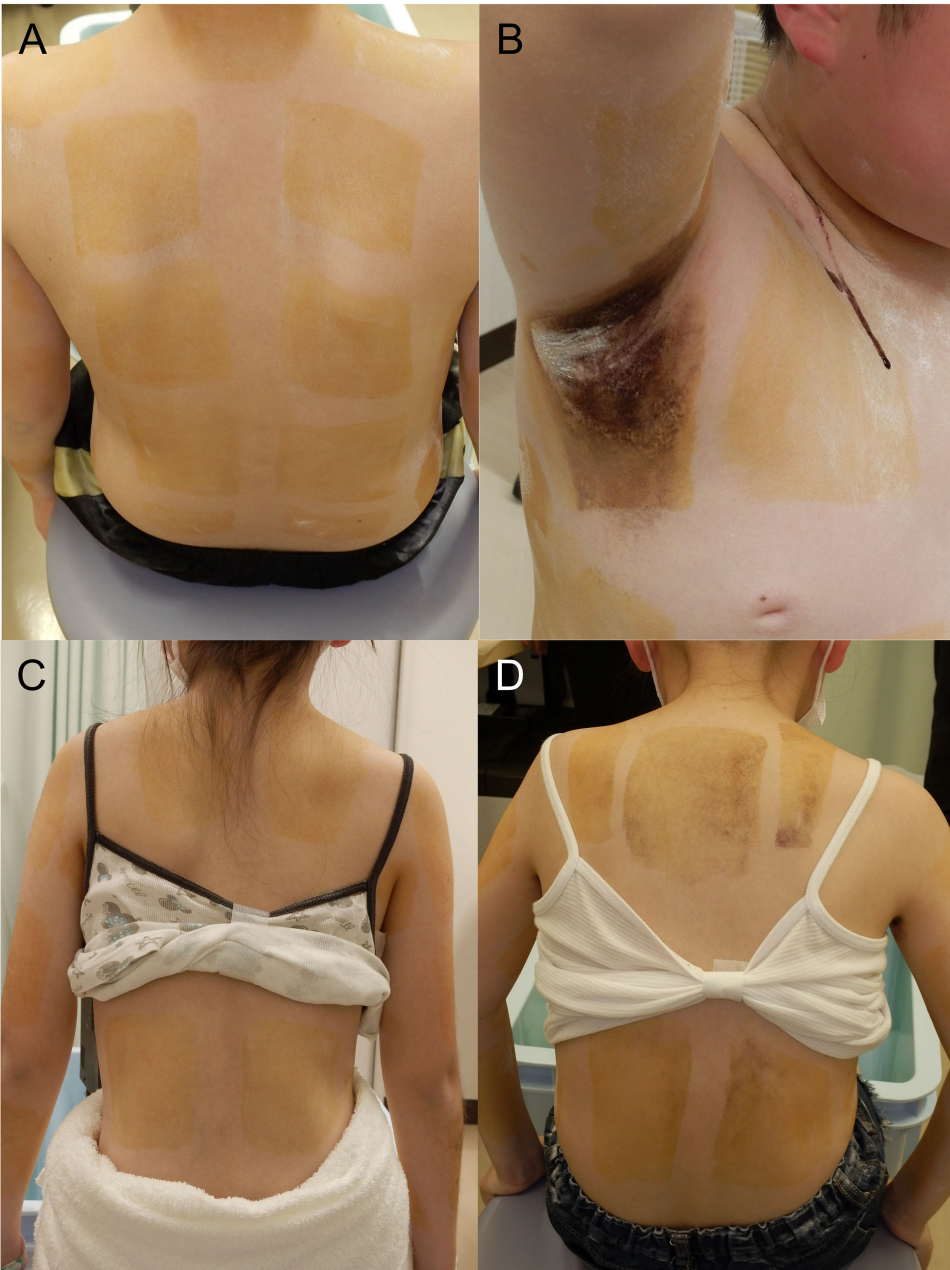
Minor’s test before **(A, C)** and after **(B, D)** chemotherapy and radiation in Patient 1 **(A, B)** and Patient 2 **(C, D)**.

**Figure 2:**
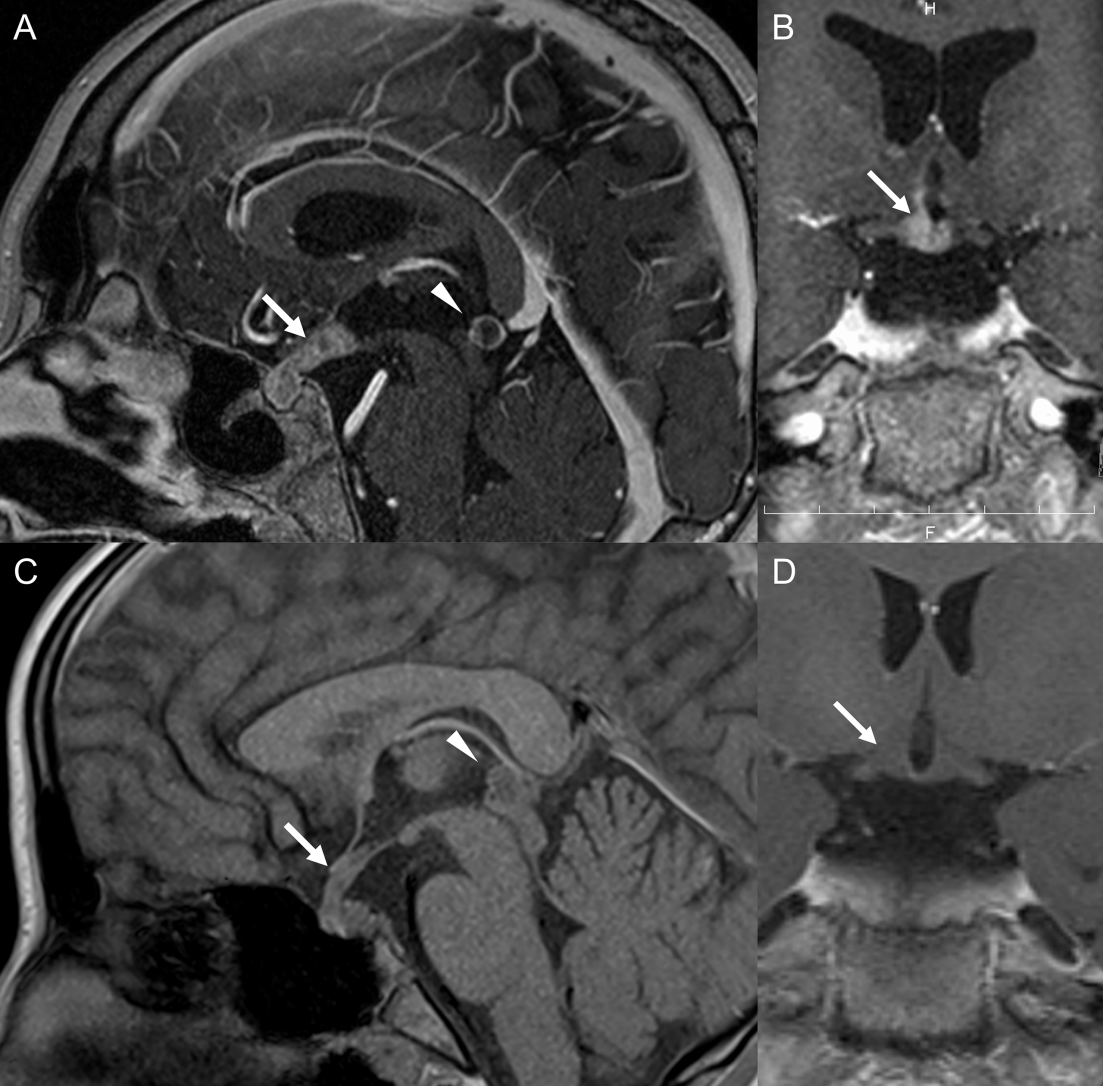
Brain gadolinium-enhanced magnetic resonance imaging (MRI) in Patient 1 **(A, B)** and Patient 2 **(C, D)**. **(A)** Arrow and arrowhead indicate an enlarged pituitary stalk and pineal body, respectively. **(B)** Arrow indicates contrasted hypothalamus, including the medial preoptic areas. **(C)** Arrow and arrowhead indicate an enlarged pituitary stalk and pineal body, respectively. **(D)** Arrow indicates that the hypothalamus was not contrasted.

A 12-year-old boy presented with repetitive heat shock-like episodes without thirst and anhidrosis for 7 months, which sometimes occurred even in winter. He had no medical or family history of endocrinological or neurological disorders. There were no symptoms with eye movement, limb movement, sensation, or reflexes. His growth curve demonstrated a decline in growth rate from a year before. He was determined to have generalized anhidrosis based on Minor’s test (**Figure 1A**). Blood examination revealed hypernatremia (161 mmol/L) and hyperosmolality (328 mOsm/kg) with decreased levels of arginine vasopressin (0.6 pg/mL; normal range, <4.0 pg/mL). Anti-SSA/SSB antibodies, the major autoantibodies in Sjögren’s syndrome (SS), were absent. Endocrinological stimulation tests revealed panhypopituitarism including deficiency of ACTH, LH/FSH, GH and TSH (**Table 1**). A skin biopsy showed no inflammation or atrophy of sweat glands. Head magnetic resonance imaging (MRI) revealed the absence of high signal intensity of the posterior pituitary. He was initially diagnosed with central diabetes insipidus (CDI) and AIGA. After the initiation of desmopressin, hypernatremia was improved, but anhidrosis persisted. A head MRI scan performed after 3 months revealed enlarged pituitary stalk and pineal body (**Figure 2A**) and gadolinium enhancement of the medial preoptic areas of the hypothalamus (**Figure 2B**). Pathological examination of the pituitary biopsy specimen revealed germinoma. After chemotherapy with carboplatin and etoposide and radiation, he continued to require hormone replacement therapy. His sweating partially resolved in the face, neck, and armpit (**Figure 1B**). At 1 year after the completion of treatment, there was no recurrence of the tumor. Due to excessive appetite, his weight increased from 30 to 60 kg in one year.

**Table 1:** Reported cases of brain tumor complicated by generalized anhidrosis at the initial evaluation.

Author, year	Age	Sex	Pathology	Tumor localization			CDI	Deficiency of anterior pituitary hormone				Complications	Clinical course of MRI	Improvement in sweating
				Pituitary	Hypo-thalamus	Pineal body		ACTH	LH/ FSH	GH	TSH			
Moon, 2005^5^	21	M	germinoma	+	+	+	+	+	+	ND	+	diplopia	The initial MRI was unremarkable; MRI at 3 months identified the tumor.	ND
Fukunaga, 2017^6^	26	M	germinoma	+	+	+	+	+	+	ND	+	fatigue, nausea	The initial MRI identified the tumor.	partial (face, palm, sole)
Kawahara, 2021 (This study)	12	M	germinoma	+	+	+	+	+	+	+	+	heat shock	The initial MRI was considered CDI; MRI at 3 months identified the tumor.	partial (face, neck, armpit)
	12	F	germinoma	+	−^*^	+	+	+	+	+	+	short stature	The initial MRI identified the tumor.	completely improved

CDI, complete diabetes insipidus; ^*^The hypothalamus was not gadolinium-contrasted and without space occupying lesion.; ND, not described.

#### 2.1.2 Patient 2

A 12-year-old girl presented with short stature, polydipsia and polyuria, and delayed puberty. She had no medical or family history of endocrinological or neurological disorders. There were no symptoms with eye movement, limb movement, sensation, or reflexes. Her growth curve revealed a decline in growth rate since 3 years before. Endocrinological stimulation tests showed panhypopituitarism including deficiency of ACTH, LH/FSH, GH and TSH (**Table 1**). Water deprivation and desmopressin test revealed complete CDI. She was determined to have generalized anhidrosis based on Minor’s test (**Figure 1C**). Even after the initiation of desmopressin, anhidrosis did not improve. Head MRI revealed the absence of high signal intensity of the posterior pituitary and enlarged pituitary stalk and pineal body (**Figure 2C**), but the hypothalamus was not gadolinium-enhanced (**Figure 2D**). Pituitary biopsy examination revealed germinoma. After chemotherapy with carboplatin and etoposide and radiation, she continued to require hormone replacement therapy. Her sweating was completely resolved (**Figure 1D**). No recurrence of the tumor was observed 6 months after the completion of treatment. She didn’t have excessive appetite.

### 2.2 Ethical Compliance

This study was approved by the Institutional Review Board Committee at the Aichi Medical University (2020-H065). Written informed consent was obtained from the parents. All study evaluations and procedures were performed in accordance with the Declaration of Helsinki.

### 2.3 Review of the Literature

#### 2.3.1 Search Strategy

We conducted a review of previous reports of brain tumor complicated by generalized anhidrosis at the initial evaluation. Case reports of patients were identified in PubMed on December 12, 2021, using the following search terms: “sweat” AND “brain tumor” (131 results); “anhidrosis” AND “central nervous system” (89 results); “hypohidrosis” AND “central nervous system” (54 results); “anhidrosis” AND “hypothalamus” (21 results); “anhidrosis” AND “brain tumor” (9 results); “hypohidrosis” AND “hypothalamus” (7 results); “hypohidrosis” AND “brain tumor” (5 results). Articles that described patients with generalized anhidrosis caused by brain tumor were retrieved and the phenotypic descriptions were analyzed. No restriction was imposed on language or date.

#### 2.3.2 Study Selection

Due to the rarity of the condition, all study designs were accepted for inclusion, including clinical and radiological studies.

#### 2.3.3 Screening and Selection of Literature

After deleting duplications, one reviewer (HI) checked all the retrieved titles, followed by abstract screening. Full manuscripts were retrieved for articles with potentially relevant abstracts.

#### 2.3.4 Data Extraction

Information related to the clinical background, tumor localization, complications of brain tumor, clinical course of MRI, and improvement in sweating was extracted for analysis from the included articles.

#### 2.3.5 Data Collection

In preparing this review, we adhered to the guidelines of the Preferred Reporting Items for Systematic Reviews and Meta Analyses 2020 statement (4).

#### 2.3.6 Data Analysis

Analysis was performed using descriptive statistics and narrative synthesis. If a space-occupying lesion or contrast effect was detected in a brain region on MRI, it was determined that there was localization of the brain tumor.

## 3 Results

### 3.1 Search Result

**Figure 3:**
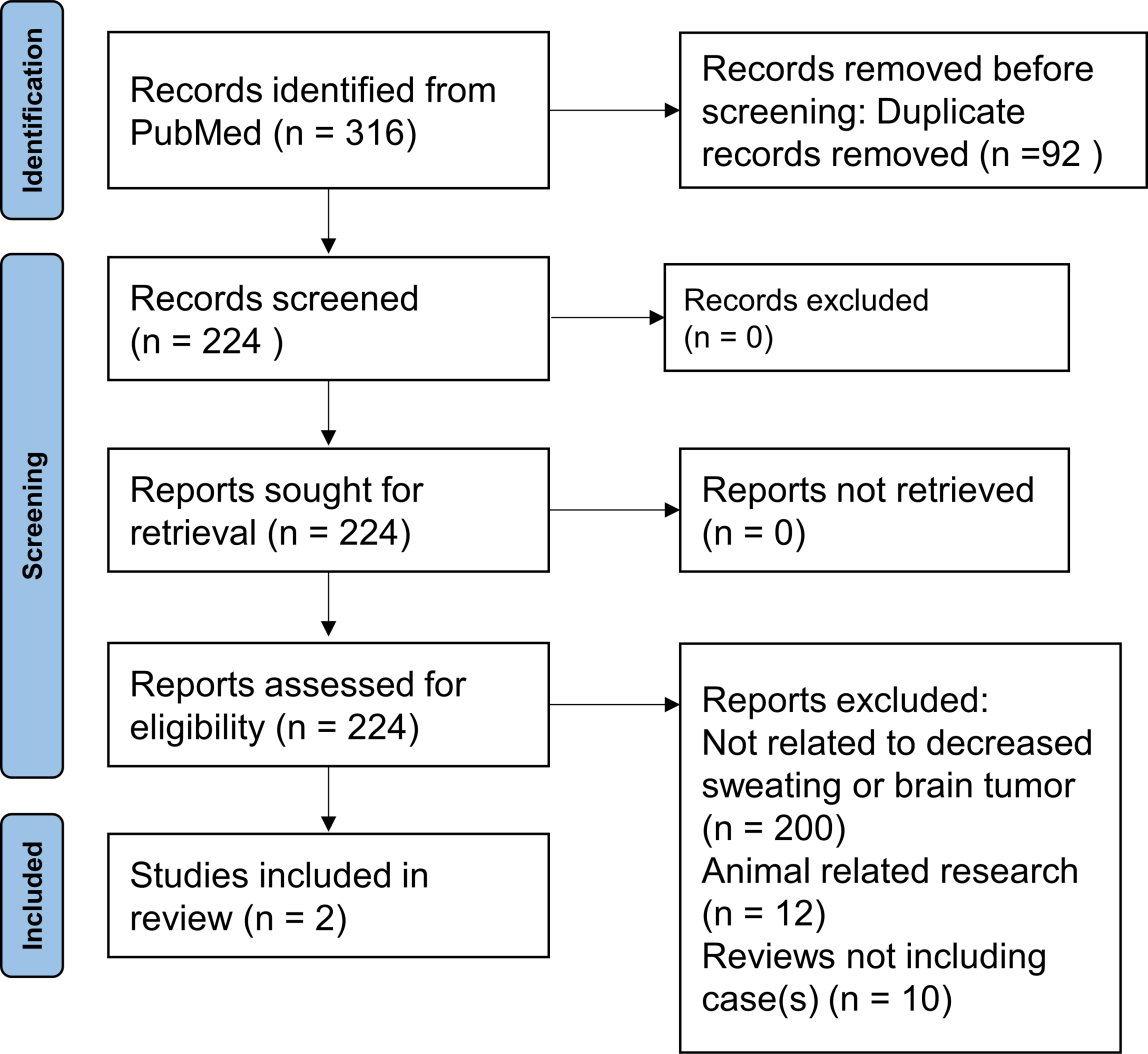
Search results of reported cases of brain tumor complicated by generalized anhidrosis at the initial evaluation.

The electronic search generated 316 hits (**Figure 3**). After deleting 92 duplicates, the initial abstract screening resulted in 224 potentially relevant articles. The number of articles and reasons for exclusion were as follows: 200 were not related to decreased sweating or brain tumor, 12 reported animal-related research, and 10 were reviews. Finally, two studies were included for analysis.

### 3.2 Demographic Results of the Pooled Samples

The pooled samples consisted of four patients with brain tumor complicated by generalized anhidrosis at the initial evaluation, including our two cases (**Table 1**) (5, 6). Their age range was 12–26 years, and three patients were men. The pathology of the brain tumors was germinoma in all patients. Three patients had tumor involvement in the pituitary, hypothalamus, and pineal gland; one patient had tumor involvement in the pituitary and pineal gland without evident hypothalamic involvement. CDI and deficiency of ACTH, LH/FSH, and TSH were observed in all patients. GH deficiency was observed in our two patients, but not described in two previously reported cases. Complications included diplopia, fatigue, nausea, heat shock, and short stature. The first MRI revealed the presence of tumors in two patients, but not in the other two patients. A subsequent MRI scan that was performed 3 months later revealed tumors in the latter two patients. Sweating was completely restored in one patient, in whom hypothalamic involvement was not evident, and partially restored in two patients. Improvement in sweating in one patient was not described.

## 4 Discussion

We report two patients with germinoma with generalized anhidrosis as the initial symptom and recovery of sweating function after treatment. In our literature review, we identified two other patients with brain tumor complicated by generalized anhidrosis as the initial symptom. Interestingly, all patients had germinoma. We speculate that damage to medial preoptic area of the hypothalamus is the cause of anhidrosis. Because medial preoptic area is considered to be a primary thermosensitive area (2).

### 4.1 Generalized Anhidrosis by Brain Tumor

Generalize anhidrosis due to brain tumors including germinoma is believed to be rare and may be easily overlooked. Although Sethi et al. reported various symptoms due to intracranial germ cell tumors in 70 pediatric patients, anhidrosis was not observed (7). Watanabe et al. reported that six of eight patients had anhidrosis after surgery for suprasellar tumors, but none of them were aware of anhidrosis (2). In addition to the rarity and unawareness of anhidrosis, it is problematic that AIGA and anhidrosis due to germinoma share similar symptoms. There are currently only 100-200 cases of AIGA reported in the literature. Most of the reported cases are from Japan. According to the guideline of AIGA in Japan (3), acquired generalized anhidrosis of unknown etiology without autonomic or neurological symptoms can be diagnosed as AIGA. There is no AIGA after brain surgery because the reason of anhidrosis is apparent. In fact, Patient 1 showed no autonomic or neurological symptoms and was initially diagnosed with AIGA. The peak age of onset of germ cell tumors is 10-30s. The fact that the four cases in this study were aged 12-26 years may reflect the peak age of germ cell tumors. Curiously, the peak age of AIGA is also in the 10-30s, and germ cell tumors may have been missed in some patients. Therefore, when treating patients with a diagnosis of AIGA, it is important to perform serial MRI evaluations to differentiate between AIGA and germinoma.

### 4.2 Recovery From Anhidrosis Due to Brain Tumor

Sweating was completely resolved in Patient 2 in whom the hypothalamus showed no post-gadolinium enhancement preoperatively. A discrepancy existed between sweating dysfunction and the absence of the gadolinium-enhanced hypothalamus. The absence of enhancement may be explained by the idea that the infiltration of germinoma was subtle in this patient, if present. This could also be related to the complete recovery of sweating function in this patient. In Patient 1, the hypothalamic dysfunction had not improved and there was excessive appetite along with continued anhidrosis; in Patient 2, there was no excessive appetite, consistent with improvement in anhidrosis. If anhidrosis is caused by decreased growth hormone or hypothyroidism, hormone replacement may improve anhidrosis, but anhidrosis did not completely improve despite tumor disappearance and hormone replacement in Patient 1 of our study and the patient reported by Fukunaga et al. Therefore, we consider it unlikely that anhidrosis was caused by hormone deficiency. Improvement of anhidrosis associated with hypothalamic lesions was also reported in a patient with neuromyelitis optica due to anti-aquaporin 4 autoantibody (8). The anhidrosis improved along with the improvement of inflammation on MRI. This indicates that anhidrosis caused by hypothalamic disorders can be reversible. Although no information of the recovery of anhidrosis caused by germinoma was obtained, these facts indicate that generalized anhidrosis caused by germinoma may completely resolve when the tumor is diagnosed earlier.

### 4.3 Limitation of This Study

The limitation of this study is the small number of cases, even including the cases in the literature review. We asked several pediatric endocrinologists and pediatric oncologists if they had ever experienced a patient with anhidrosis at the time of initial diagnosis of brain tumor. However, none of the physicians performed sweating tests at the time of initial diagnosis, nor did they experience any patients with anhidrosis. Therefore, patients with hypothalamic and/or pituitary tumors should be evaluated for anhidrosis by sweating tests. In order to accumulate cases with anhidrosis at the time of initial diagnosis of brain tumor, a nationwide survey is planned in the future.

## 5 Conclusion

Generalized anhidrosis could be a rare but important initial symptom of germinoma and may resolve when the tumor is diagnosed earlier. Germinoma should be considered even when no obvious lesion is observed at the first presentation and a head MRI should be performed repetitively.

## Data Availability Statement

The original contributions presented in the study are included in the article/supplementary material. Further inquiries can be directed to the corresponding author.

## Ethics Statement

The studies involving human participants were reviewed and approved by the Institutional Review Board Committee at the Aichi Medical University. Written informed consent to participate in this study was provided by the participants’ legal guardian/next of kin. Written informed consent was obtained from the individual(s), and minor(s)’ legal guardian/next of kin, for the publication of any potentially identifiable images or data included in this article.

## Author Contributions

KK contributed to the study design, interpretation of data, and was a major contributor in writing the manuscript. YS, YO, TW, and TH contributed to the acquisition and analysis of data from the patients. AO and HI revised the manuscript critically for important intellectual content. All authors read and approved the final manuscript. All authors approved the final manuscript as submitted and agree to be accountable for all aspects of the work.

## Funding

The research of HI was supported by JSPS KAKENHI, Grant No. 21K07783. The research of AO was supported by JSPS KAKENHI, Grant No. 21K07810.

## Conflict of Interest

The authors declare that the research was conducted in the absence of any commercial or financial relationships that could be construed as a potential conflict of interest.

## Publisher’s Note

All claims expressed in this article are solely those of the authors and do not necessarily represent those of their affiliated organizations, or those of the publisher, the editors and the reviewers. Any product that may be evaluated in this article, or claim that may be made by its manufacturer, is not guaranteed or endorsed by the publisher.
